# Regioselectivity of the S_E_Ar-based cyclizations and S_E_Ar-terminated annulations of 3,5-unsubstituted, 4-substituted indoles

**DOI:** 10.3762/bjoc.18.33

**Published:** 2022-03-08

**Authors:** Jonali Das, Sajal Kumar Das

**Affiliations:** 1 Department of Chemical Sciences, Tezpur University, Napaam, Tezpur-784028, Assam, India

**Keywords:** annulation, cyclization, fused indoles, regioselectivity, S_E_Ar

## Abstract

Indole-3,4- and 4,5-fused carbo- and heterocycles are ubiquitous in bioactive natural products and pharmaceuticals, and hence, a variety of synthetic approaches toward such compounds have been developed. Among these, cyclization and annulation of 3,5-unsubstituted, 4-substituted indoles involving an electrophilic aromatic substitution (S_E_Ar) as the ring closure are particularly attractive, because they avoid the use of 3,4- or 4,5-difunctionalized indoles as starting materials. However, since 3,5-unsubstituted, 4-substituted indoles have two potential ring-closure sites (indole C3 and C5 positions), such reactions in principle can furnish either or both of the indole 3,4- and 4,5-fused ring systems. This Commentary will briefly highlight the issue by summarizing recent relevant literature reports.

## Introduction

**Scheme 1:**
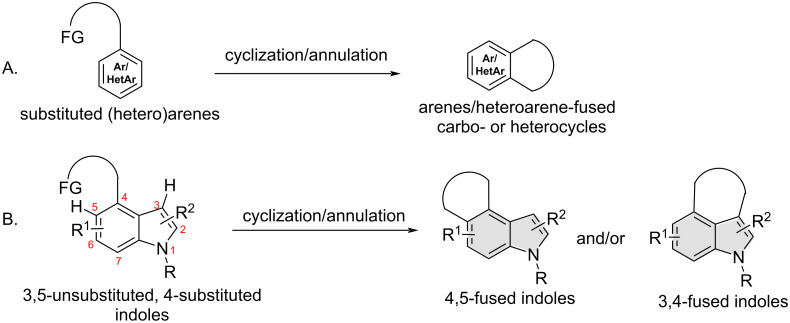
S_E_Ar-based, C_Ar_–C bond-forming cyclization or annulation of: (A) substituted arenes/heteroarenes and (B) 3,5-unsubstituted, 4-substituted indoles.

Over the decades, countless cyclization and annulation reactions of substituted arenes/heteroarenes involving an electrophilic aromatic substitution (S_E_Ar) reaction as the ring-closure step have been routinely employed for the construction of diverse arene- and heteroarene-fused rings (Scheme 1A) [1–3]. In most of these approaches, the new C_Ar_–C bond is formed *ortho* to the tether/directing functionality on an aromatic or a heteroaromatic ring, as the geometrical constraints do not normally allow *meta* or *para*-selective cyclization/annulation. In certain such cyclization and annulation reactions, however, formation of the C_Ar_–C bond at the *ortho* position is not guaranteed. As an example, cyclization and annulation of 3,5-unsubstituted, 4-substituted indoles involving an S_E_Ar reaction as the ring-closure step can generate indole 3,4-fused cabo- and heterocycles or/and their indole 4,5-fused counterparts (Scheme 1B). This is primarily due to the fact that such substrates have two proximal nucleophilic sites: the indole C5 as the *ortho* position and intrinsically highly nucleophilic indole C3 as the *peri* position, with the latter being often more nucleophilic than the former. Noteworthy is that the kinetic preference for ring-closure onto the C5 position may be a more dominating factor than the higher nucleophilicity of the C3 position (or vice versa).

Due to this regiochemical uncertainty, it is no surprise that the scope of cyclization and annulation of 3,5-unsubstituted, 4-substituted indoles has not been studied to the same extent as the scope of the cyclization and annulation of *N*-, 2-, or 3-substituted indoles [4]. However, several synthetically attractive cyclization and annulation of 3,5-unsubstituted, 4-substituted indoles have been reported in the recent past. Herein, we summarize these literature reports, with a special attention on the regiochemistry. Noteworthy is that although three reviews on the synthesis of 3,4-fused indoles have been published in the last decade [5–7], this is the first time the regioselectivity of the S_E_Ar-based/terminated cyclization and annulation reactions of 3,5-unsubstituted, 4-substituted indolesis is sytematized as a dedicated topic.

## Discussion

**Scheme 2:**
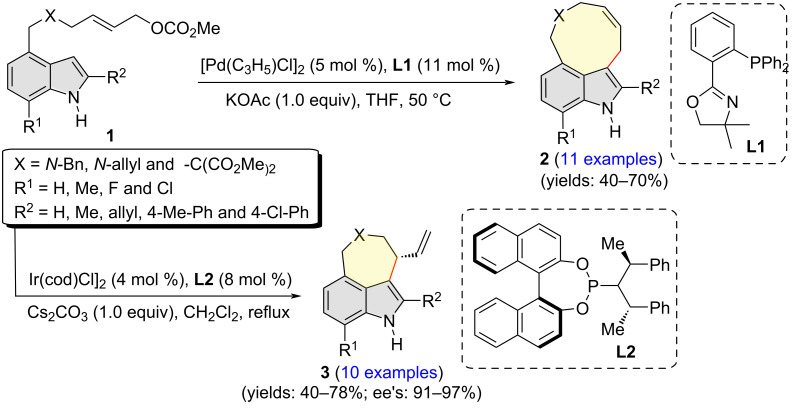
Indole C3 regioselective intramolecular alkylation of indolyl allyl carbonates.

The Tsuji–Trost reaction serves as a powerful tool in constructing carbon–carbon and carbon–heteroatom bonds in organic synthesis [8–10]. In the course of their diversity-oriented synthesis of indole-based *peri*-annulated compounds, You and co-workers in 2013 reported the intramolecular Tsuji–Trost reaction of indolyl allyl carbonates **1** under the catalysis of [Pd(C_3_H_5_)Cl]_2_ and ligand **L1** (Scheme 2) [11]. The reaction, that could also be considered as Friedel–Crafts type, intramolecular allylic alkylation, delivered nine-membered ring bearing 3,4-fused indoles **2** in moderate to good yields. In the asymmetric version of the reaction catalyzed by [Ir(cod)Cl]_2_ (4 mol %) and ligand **L2**, the ring closure also took place regioselectively at the indole C3 position, albeit the products **3** were embedded with a seven-membered ring instead of a nine-membered one. The absolute configuration of products **3** was proposed to be *S*, based on the general rule of stereochemistry in the Ir-catalyzed allylic substitution reactions.

**Scheme 3:**
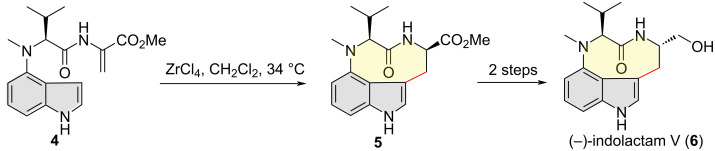
Indole C3 regioselective Michael-type cyclization in the total synthesis of (−)-indolactam V.

In 2016, Billingsley and co-workers disclosed the total synthesis of (−)-indolactam V (**6**), a nanomolar agonist of protein kinase C (Scheme 3) [12]. The authors applied an intramolecular S_E_Ar reaction of 4-substituted indole derivative to construct a 3,4-fused tricyclic indole in a late stage of their total synthesis. Specifically, Michael-type addition of compound **4** took place regio- and diastereoselectively at the indole C3 position, furnishing tricyclic compound **5** (77%) which was then elaborated into the target natural product **6** in two steps.

**Scheme 4:**
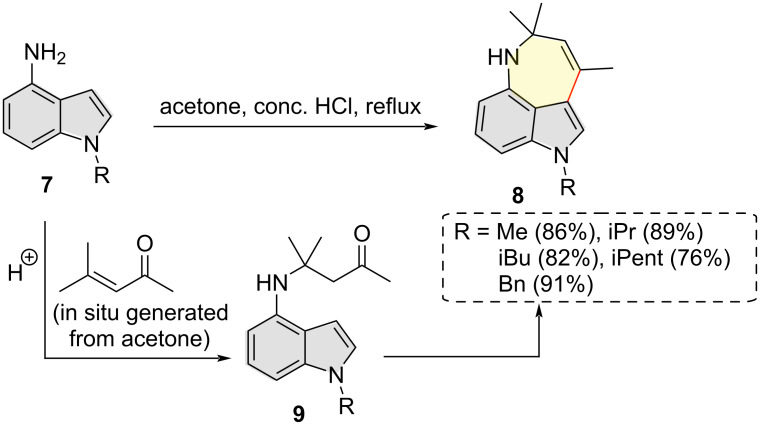
Synthesis of azepino[4,3,2-*cd*]indoles via indole C3 regioselective aza-Michael addition/cyclization/dehydration sequence.

In 2017, Lesyk and co-workers observed the indole C3 regioselective ring closure in the reaction between 4-aminoindoles **7** and acetone in the presence of hydrochloric acid as a catalyst (Scheme 4) [13]. Based on NMR spectroscopy and X-ray crystallographic analysis, the products were unambiguously assigned as 1-alkyl-3,5,5-trimethyl-5,6-dihydro-1*H*-azepino[4,3,2-*cd*]indoles **8**. The authors proposed that aza-Michael addition of 4-aminoindoles **7** to in situ generated mesityl oxide gives compound **9** which undergoes a regioselective intramolecular cyclization–dehydration sequence to furnish **8**.

**Scheme 5:**
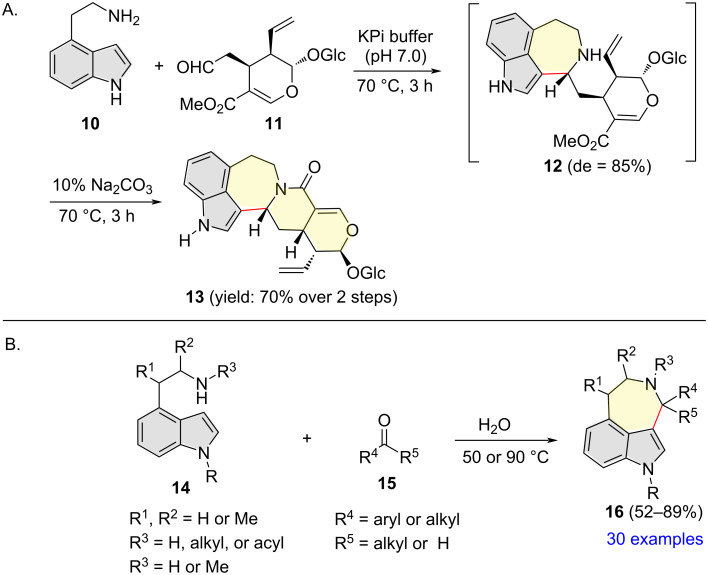
Indole C3 regioselective Pictet−Spengler reaction of 2-(1*H*-indol-4-yl)ethanamines.

In 2019, Zou and co-workers reported that the ring closure of the Pictet−Spengler reaction between 2-(1*H*-indol-4-yl)ethanamine (**10**) and secologanin (**11**) in potassium phosphate buffer (KPi) at 70 °C regioselectively took place at the indole C3 position, resulting in unstable 4β-(*R*)-1*H*-azepino[3,4,5-*cd*]indolylvincoside **12** (de = 85%) [14]. In situ lactamization of **12** under basic conditions (10% Na_2_CO_3_) generated stable polycyclic compound **13** in 70% yield (Scheme 5A). In a separate report published in 2020, the same research group disclosed that water could act as both catalyst and solvent in the Pictet−Spengler reaction of 2-(1*H*-indol-4-yl)ethanamines **14** with various aldehydes/ketones **15**, delivering a variety of azepino[3,4,5-*cd*]indoles **16** in a straightforward fashion in moderate to high yields (Scheme 5B) [15]. The reaction tolerated various alkyl and aryl aldehydes and dialkyl ketones, irrespective of their electronic nature. Environment-friendly reaction conditions, easily accessible substrates, and broad substrate scope highlight the practicality of this methodology.

**Scheme 6:**
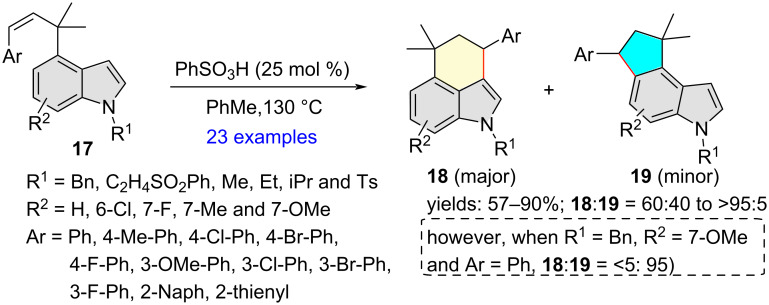
Indole C3 regioselective hydroindolation of *cis*-β-(α′,α′-dimethyl)-4′-methindolylstyrenes.

Results of the acid-catalyzed intramolecular hydroindolation of *cis*-β-(α′,α′-dimethyl)-4′-methindolylstyrenes **17** were reported by Stokes and co-workers in 2019 (Scheme 6) [16]. The authors observed that **17** could be cyclized under PhSO_3_H catalysis in toluene at 130 °C to tetrahydrobenzo[*cd*]indoles **18** in 57–90% yields as major products, together with minor amounts of the corresponding tetrahydrocyclopenta[*e*]indoles **19** (**18**:**19** = 60:40 to >95:5). In majority of the cases, compounds **18** could be purified from the regioisomeric mixtures. Notably, no reaction took place with a *N*-acetyl-protected substrate, and a free N−H indole substrate decomposed. Furthermore, introduction of an electron-donating OMe group at the indole 7 position reversed the regioselectivity in favor of the 4,5-fused indole system. Based on their experimental and computational investigations, the researchers hypothesized that Thorpe–Ingold effect could induce dispersive interactions between the indole and styrene moieties, triggering the preferential formation of the 3,4-fused indoles **18** via a concerted protonation and C–C bond formation. Weakened dispersive interactions caused by a substituent or heteroatom resulted in low yields and reduced regioselectivities.

**Scheme 7:**
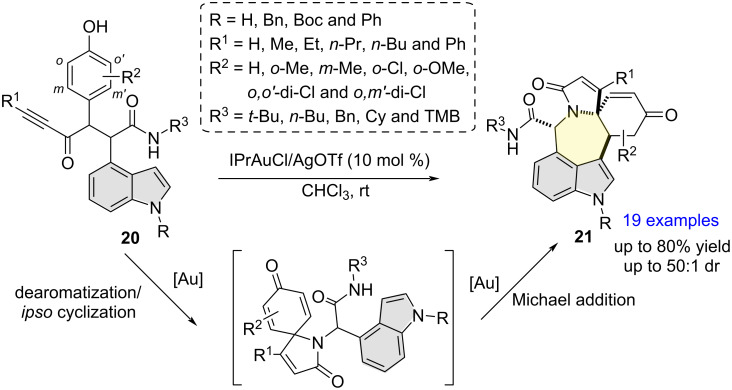
Indole C3 regioselective cyclization leading to the formation of polycyclic azepino[5,4,3-*cd*]indoles.

In 2020, Li and Van der Eycken and co-workers reported the synthesis of densely functionalized, polycyclic azepino[5,4,3-*cd*]indoles **21** from the intramolecular cyclization of Ugi adducts **20** in moderate to good yields and excellent chemo-, regio-, and diastereoselectivity (Scheme 7) [17]. Mechanistically, the reaction involves a tandem gold(I)-catalyzed dearomatization/*ipso*-cyclization/Michael addition sequence to substrates **20**. Noteworthy is that substrates bearing an indolyl N–Ph or N–Boc moiety (instead of free indolyl N–H) failed to deliver the corresponding final cyclized products. The authors attributed this failure to the indole C3 position’s reduced nucleophilicity which thwarted the Michael addition step.

**Scheme 8:**
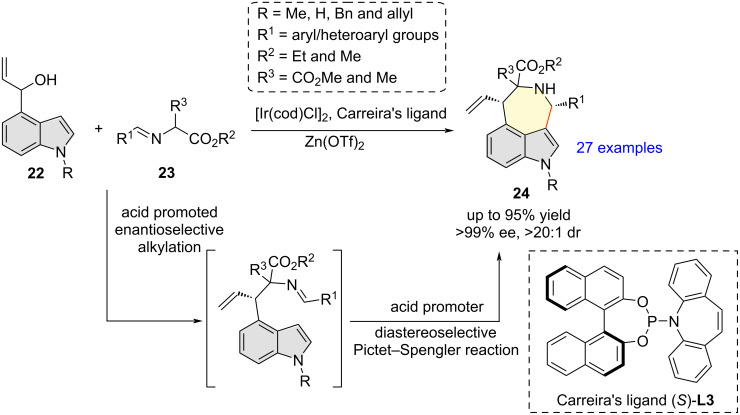
Synthesis of azepino[3,4,5-*cd*]indoles via iridium-catalyzed asymmetric [4 + 3] cycloaddition of racemic 4-indolyl allylic alcohols with azomethine ylides.

In 2021, Deng et al. showcased an unprecedented iridium-catalyzed asymmetric [4 + 3] cycloaddition of racemic 4-indolyl allylic alcohols **22** with α-imino esters **23** as azomethine ylide precursors to afford azepino[3,4,5-*cd*]indoles **24** in good yields and with complete regioselectivity and generally excellent diastereo- and enantioselectivities (up to >20:1 dr and >99% ee) (Scheme 8) [18]. The optimized reaction conditions for the annulation reaction were as follows: [Ir(cod)Cl]_2_ (4 mol %), Carreira’s P/olefin ligand (*S*)-**L3** (16 mol %), Zn(OTf)_2_ (100 mol %), and 4 Å MS in CH_2_Cl_2_ at rt. The synthetic protocol tolerates a variety of substituents in both **22** and **23**. From a mechanistic point of view, the reaction proceeds through a domino azomethine ylide formation/allylation/Pictet–Spengler reaction sequence.

**Scheme 9:**
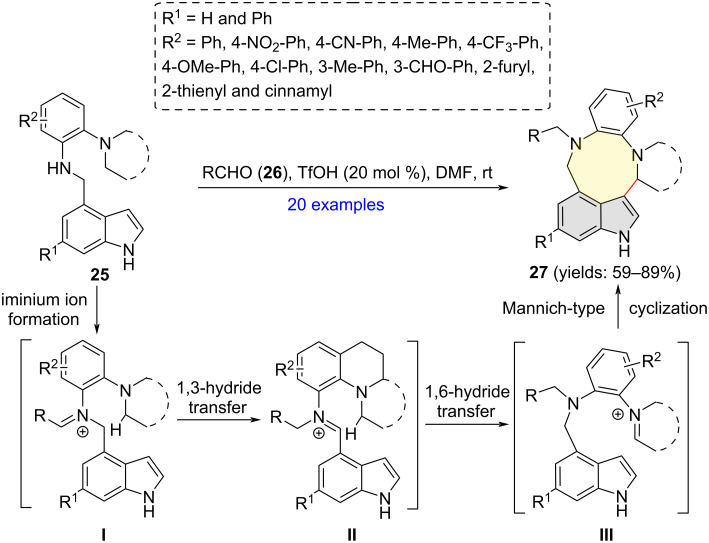
Aldimine condensation/1,6-hydride transfer/Mannich-type cyclization cascade of indole-derived phenylenediamines.

Recently, An and Xiao and co-workers disclosed high-yielding syntheses of a wide range of indole-3,4-fused nine-membered rings **27** via triflic acid (TfOH)-catalyzed reaction of indole-derived phenylenediamine **25** with aldehydes **26** (Scheme 9) [19]. Mechanistically, the initially formed iminium ion **I** undergoes isomerization to iminium ion **II** through a 1,3-hydride shift process. Iminium ion **III** could then be generated via 1,6-hydride shift in both **I** and **II**. Finally, an intramolecular Mannich-type cyclization then furnishes products **27**. The cascade protocol enjoys several advantageous synthetic features, including high step- and atom-economy, transition-metal-free and room temperature conditions.

**Scheme 10:**
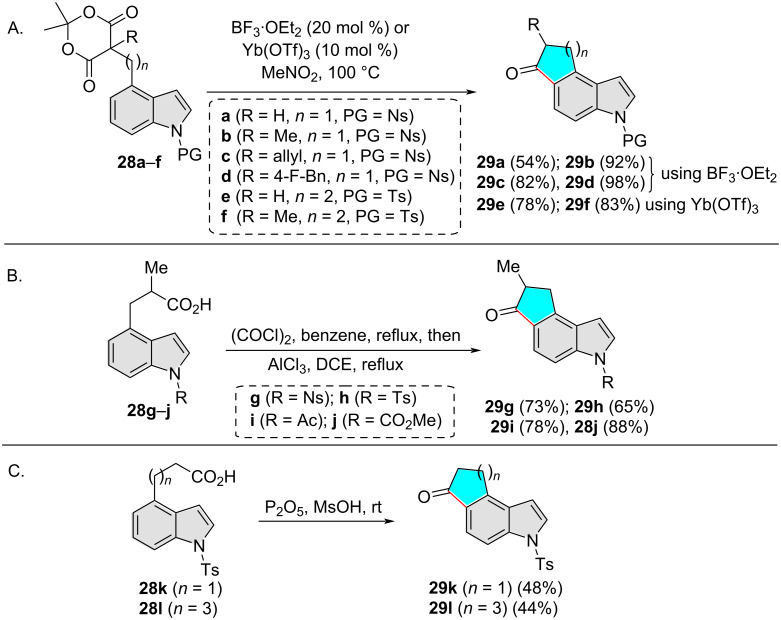
Indole C5 regioselective intramolecular FC acylation of 4-substituted indoles.

In all cases shown above, the new C_Ar_–C bond is exclusively formed at the indole C3 position. However, cyclization of 4-substituted indoles that takes place regioselectively at the indole C5 positions has also been reported, albeit on rare occasions. One of the first such reports was disclosed by Dumas and Fillion in their studies on the intramolecular Friedel−Crafts (FC) acylation of 4-substituted indoles [20]. Specifically, *N*-Ns/Ts-indolyl Meldrum's acid derivatives **28a**–**f** delivered 4,5-fused indoles **29a**–**f** under BF_3_·OEt_2_ or Yb(OTf)_3_ catalysis (Scheme 10A). It should be noted that the authors could not extend this methodology to related substrates with free indolyl NH as such reactions resulted into a complex mixture (not shown here), possibly due to the decomposition of the substrates. Nevertheless, the same regioselectivity was observed when the FC-acylation of *N*-protected 3-(4-indolyl)propanoic acids **28g**–**j** was performed by converting them into the corresponding acid chloride, followed by treatment with AlCl_3_ in refluxing 1,2-dichloroethane (DCE) (Scheme 10B). The reactions furnished indole 4,5-fused indanones **29g**–**j** as the only detectable cyclized products. The authors noted that the kinetic preference for cyclization onto the C5 position is more dominating than the higher nucleophilicity of the C3 position, making the C_Ar_–C bond formation completely regioselective at the C5 position. Subsequently in 2017, Li and co-workers also applied the intramolecular Friedel–Crafts acylation strategy to get cyclopenta[*e*]indol-6-one **29k** and cyclohepta[*e*]indol-6-one **29l** from compounds **28k** and **28l**, respectively (Scheme 10C) [21].

**Scheme 11:**
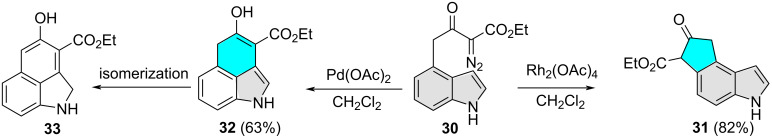
Catalyst-dependent regioselectivity switching in the cyclization of ethyl 2-diazo-4-(4-indolyl)-3-oxobutanoate.

In 2009, the Hansen group disclosed that treatment of 2-diazo-4-(4-indolyl)-3-oxobutanoate **30** with a catalytic amount of Rh_2_(OAc)_4_ resulted in the formation of the 4,5-fused tricyclic indole derivative **31** in 82% yield (Scheme 11) [22]. Being a kinetically very active catalyst, Rh_2_(OAc)_4_ favored the formation of the five-membered ring. On the other hand, employment of Pd(OAc)_2_-catalysis switched the regioselectivity of this C–H insertion reaction. More specifically, under Pd(OAc)_2_ catalysis diazo compound **30** delivered 3,4-fused tricyclic indole derivative **32** which underwent spontaneous rearrangement to thermodynamically more stable naphthalene derivative **33** upon standing for a few hours. To the best of our knowledge, this is the only report of catalyst-controlled C3 versus C5 regioselectivity switching in the S_E_Ar-based cyclizations of 3,5-unsubstituted, 4-substituted indoles.

**Scheme 12:**
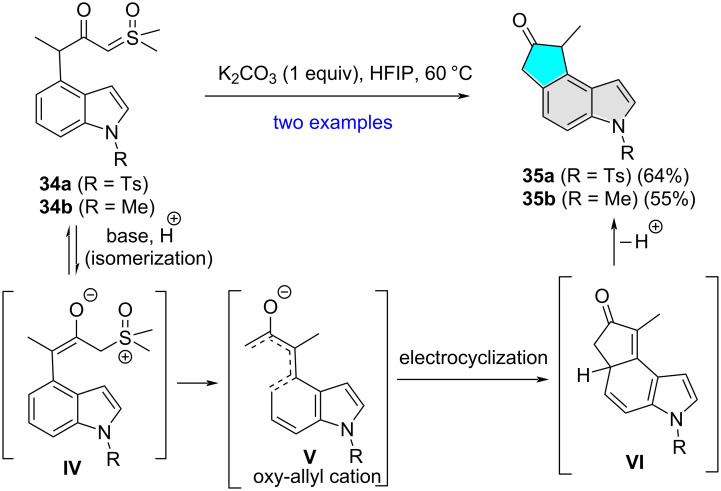
Indole C5 regioselective cyclization of α-carbonyl sulfoxonium ylides.

In the course of their studies on chemospecific cyclization of α-carbonyl sulfoxonium ylides on aryls and heteroaryls, the Aïssa group in 2019 demonstrated hexafluoroisopropanol (HFIP)-promoted regioselective cyclization of β-carbonyl sulfoxonium ylides **34a**,**b** in the presence of K_2_CO_3_ to access cyclopenta[*e*]indol-6-ones **35a**,**b** in moderate yields (Scheme 12) [23]. The authors proposed that under the experimental conditions β-carbonyl sulfoxonium ylides **34a**,**b** were isomerized to **IV** which then produced oxy-allyl cation **V**. Electrocyclization of **V** followed by loss of proton from the intermediate **VI** afforded the corresponding cyclopenta[*e*]indol-6-ones **35a**,**b**.

**Scheme 13:**
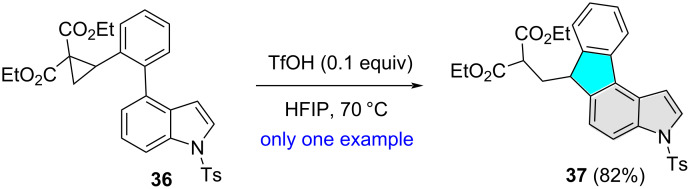
Indole C5 regioselective cyclization of an indole-tethered donor–acceptor cyclopropane.

While involved in the synthesis of 9*H*-fluorenes and 9,10-dihydrophenanthrenes through intramolecular arylative ring-opening of indole-tethered donor–acceptor cyclopropanes, Li and co-workers treated compound **36** with triflic acid (TfOH) in refluxing HFIP (Scheme 13) [24]. The reaction afforded compound **37** in 82% through the regioselective intramolecular ring-opening of the cyclopropane ring at the benzylic carbon atom.

**Scheme 14:**
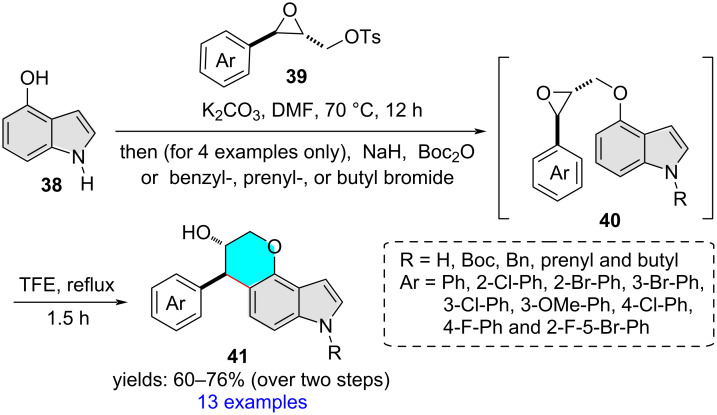
Indole C5 regioselective epoxide–arene cyclization.

Very recently, our group has reported the synthesis of pyrano[2,3-*e*]indol-3-ols **41** via trifluoroethanol-mediated intramolecular ring-opening cyclization of 4-(2-oxiranylmethoxy)indoles **40** which were prepared by *O*-alkylation of 4-hydroxyindole **38** using epoxy tosylates **39** as the alkylating agents, followed by (in selected cases) *N*-*tert*-butyloxycarbonylation and *N*-alkylation (Scheme 14) [25]. The C5 cyclization regioselectivity and *trans*-diastereoselectivity were not influenced by the electronic nature of the indole-*N*-substituent.

## Conclusion

As illustrated by these studies, S_E_Ar-based intramolecular cyclization and annulation reactions of 3,5-unsubstituted, 4-substituted indoles have recently been successfully applied in the construction of indole 3,4- and 4,5-fused carbo- and heterocycles. Notably, most of these reactions are highly to completely regioselective, despite having two potential ring-closure sites in the substrates. From these reports, some trends in the prediction of site of ring-closure can be derived. In general, absence of an electron-donating group in the indole benzene ring promotes C3 regioselective cyclization, provided the size of the newly formed ring is greater than six. Under the opposite scenario, C5 regioselective cyclization is observed. Moreover, the use of the electron-withdrawing protecting group on the indole N atom could favor the formation of 4,5-fused indoles by decreasing the nucleophilicity at the indole C3 position. However, detailed studies of the effect of the electronic nature of the indole NH protecting group on the regioselectivity are yet to be reported for S_E_Ar-based intramolecular cyclization and annulation reactions of 3,5-unsubstituted, 4-substituted indoles. Such studies in this area will certainly aid in elucidating the regioselectivity more precisely.
